# A Case of Bilateral Testicular Tumors Subsequently Diagnosed as Congenital Adrenal Hyperplasia Due to 21-Hydroxylase Deficiency 

**DOI:** 10.22074/ijfs.2015.4618

**Published:** 2015-12-23

**Authors:** Yan-Kun Sha, Yan-Wei Sha, Lu Ding, Wei-Wu Liu, Yue-Qiang Song, Jin Lin, Xue-Mei He, Ping-Ping Qiu, Ling Zhang, Ping Li

**Affiliations:** 1 Department of Nephrology, First Affiliated Hospital of Liaoning Medical University, Jinzhou City 121000, Liaoning Province, China; 2 Reproductive Medicine Center, Maternal and Child Health Hospital of Xiamen City 361005, Xiamen City, Fujian Province, China; 3 Department of Radiation, The Second Hospital of Jilin University, Changchun City 130041, Jilin Province, China

**Keywords:** 21-hydroxylase Deficiency, Congenital Adrenal Hyperplasia, Precocious Puberty

## Abstract

21-hydroxylase deficiency (21-OHD) caused congenital adrenal hyperplasia (CAH) is a group of autosomal recessive genetic disorders resulting from mutations in genes involved with cortisol (CO) synthesis in the adrenal glands. Testicular adrenal rest tumors (TARTs) are rarely the presenting symptoms of CAH. Here, we describe a case of simple virilizing CAH with TARTs, in a 15-year-old boy. The patient showed physical signs of precocious puberty. The levels of blood adrenocorticotropic hormone (ACTH), urinary 17-ketone steroids (17-KS), dehydroepiandrosterone sulfate (DHEA-S), and serum progesterone (PRGE) were elevated, whereas those of follicle-stimulating hormone (FSH), luteinizing hormone (LH), and CO were reduced. Computed tomography (CT) of the adrenal glands and magnetic resonance imaging (MRI) of the testes showed a soft tissue density (more pronounced on the right side) and an irregularly swollen mass (more pronounced on the left side), respectively. Pathological examination of a specimen of the mass indicated polygonal/circular eosinophilic cytoplasm, cord-like arrangement of interstitial cells, and lipid pigment in the cytoplasm. Immunohistochemistry results precluded a diagnosis of Leydig cell tumors. DNA sequencing revealed a hackneyed homozygous mutation, I2g, on intron 2 of the *CYP21A2* gene. The patient’s symptoms improved after a three-month of dexamethasone therapy. Recent radiographic data showed reduced hyperplastic adrenal nodules and testicular tumors. A diagnosis of TART should be considered and prioritized in CAH patients with testicular tumors. Replacement therapy using a sufficient amount of dexamethasone in this case helps combat TART.

## Introduction

Congenital adrenal hyperplasia (CAH) comprises a group of autosomal recessive genetic disorders affecting cortisol (CO) synthesis in the adrenal glands (1). As a result, there is a compensatory increase in the secretion of adrenocorticotropic hormone (ACTH), resulting in adrenal hyperplasia. Worldwide, the incidence of CAH in newborns is approximately 1/16,000–1/20,000, and approximately 1/15,000–1/16,000 in Europe and the USA. In China, the incidence of CAH is unknown due to the absence of a national screening program (2).

Deficiencies in 21-hydroxylase (21-OH), 11β2 hydroxylase, 3β2 steroid dehydrogenase, and 17α2 hydroxylase lead to CAH. Of these, 21-OH deficiency (21-OHD) is the most common, accounting for 90-95% of pediatric CAH cases. There are classic and non-classic CAH presentations, reflecting the extent of 21-OHD (3). Classic CAH includes the simple virilizing and salt-wasting types. Among these, simple virilizing CAH is caused by partial 21-OHD, resulting in increased androgen levels, without salt wasting. Clinically, boys with this type of CAH may show pseudo-precocious puberty. Excessive androgen secretion may also inhibit the release of pituitary gonadotropin, leading to spermatogenic disorders and fertility deficits.

The formation of adrenal glands and gonads is initiated via a common primordium, which develops into different tissues during embryogenesis, with adrenal cells possibly being transferred into the XY gonad during differentiation (4). Residual adrenal cells are reported to exist in the testes of at least 15% of healthy, newborn babies (5). The presence of abnormal, residual adrenal cells leads to the development of testicular adrenal rest tumors (TARTs). Due to insufficient endocrine regulation, the compensatory secretion of ACTH from the pituitary may reach a level high enough to cause hypertrophy and hyperplasia of the testicular adrenal-derived cells. Subsequently, TARTs may worsen regulation disorders of the hypothalamic- pituitary-adrenal axis (6). In fact, TARTs are not rare in CAH cases, especially in classic CAH, and may also be found in individuals with nonclassic CAH (7). However, TARTs, accompanying untreated CAH, may be somewhat difficult to diagnose and treat. Some doctors might misdiagnose them as malignancies and recommend surgical resection, potentially causing irreparable damage to the patient. Therefore, to help raise understanding of this condition and improve its management, we describe our experience with a case of bilateral TARTs, subsequently diagnosed as 21-OHD-related, simple virilizing CAH in a 15-year-old boy.

## Case report

The research and report are approvaled by both the patient and the Ethical Committee of our hospital.

A 15-year-old boy, one of our outpatients, was diagnosed with bilateral testicular tumors. Upon presentation, his blood pressure was 117/81 mmHg, and he was 142-cm tall. His skin was deeply pigmented, and the oral mucosa, areolas, and genitalia were also pigmented; he also demonstrated convex laryngeal tuberculosis and whiskers around his lips. Physical examination revealed the length of his penis to be approximately 9.5 cm, and he had type IV pubic hair distribution, according to the Tanner grading system. A palpable lump, 2.0×1.6×1.2 cm in size, was found on his right testis (testicular volume, 12 mL) and another, 2.5×2.0×1.8 cm in size, on his left testis (testicular volume, 20 mL). Both lumps were indurated, with irregular surfaces and had normal mobility. The patient’s prostate appeared normal.

A thorough medical history revealed that our patient was born full term (birth weight, 3.40 kg), but had experienced birth trauma. The patient’s physique had always been unsatisfactory, with some secondary sexual characteristics such as a lower voice tone, larger penis/scrotum, and pubic hair developing around three years of age. Three to four years later, his physical stature had been considerably larger than that of his peers, and his secondary sexual characteristics were more pronounced. His condition was diagnosed as sexual precocity at another hospital, but it was never treated.

In order to confirm the etiology and origin of the disease, we conducted the recommended tests for diagnosing CAH. An ACS 180 SE chemiluminescence analyzer (Bayer Diagnostics, USA) was used to determine his blood levels of ACTH, CO (1), aldosterone (ALD), and a series of tumor markers [alpha-fetoprotein, carbohydrate antigen (CA), prostate antigen, and carcinoembryonic antigen (CEA)]. After the detection of ACTH and CO levels, we administered low-dose dexamethasone (0.75 mg, orally, every 6 hours, for 3 days) and redetermined his levels of ACTH and CO (Table 1).

Another chemiluminescence analyzer (Access, Beckman Coulter, USA) was used to check the patient’s levels of sex hormones, including folliclestimulating hormone (FSH), luteinizing hormone (LH), prolactin (PRL), progesterone (PRGE), estradiol (E_2_), testosterone (T), human chorionic gonadotropin (hCG), and dehydroepiandrosterone sulfate (DHEA-S). After administering low-dose dexamethasone, the DHEA-S level was re-calculated (Table 2).

A CX4 automatic biochemical apparatus (Beckman Coulter) was used to detect urinary levels of 17-ketone steroids (17-KS), 17-hydroxyl steroids (17-OH), and vanillaalmond acid (VMA). To test liver/kidney function, the Bayer-500 (Bayer Diagnostics) urine analyzer was used to analyze the patient’s urine (Table 3).

**Table 1:** Levels of adrenocorticotropic hormone (ACTH) and cortisol (CO)


Time	First detection		Second detection (after dexamethasone suppression)		Reference range

8:00	ACTH 1250 pg/mL	CO 79.53 nmol/L	ACTH 736 pg/mL	CO 24.6 nmol/L	ACTH: 0–46 pg/mL CO: 118.6–618 nmol/L
16:00	ACTH 971 pg/mL	CO 66.39 nmol/L	ACTH 110.00 pg/mL	CO 93.77 nmol/L	ACTH: 0–46 pg/mL CO: 85.3–459.6 nmol/L
0:00 (the following day)	ACTH 423.00 pg/mL	CO 39.13 nmol/L	ACTH 51.4 pg/mL	CO 2.39 nmol/L	ACTH: 0–46 pg/mL CO: 118.6–618 nmol/L


**Table 2:** Levels of sex hormones


Time	Level	Reference range

FSH	0.56 mIU/mL	1.4–18.1 mIU/mL
LH	0.01 mIU/mL	1.5–9.3 mIU/mL
PRL	10 ng/mL	2.1–17.7 ng/mL
PRGE	27.27 pg/mL	0.28–1.22 pg/mL
E2	9.93 pg/mL	0–52 pg/mL
T	755.84 ng/dL	241–827 ng/mL
hCG	0.0 mIU/mL	0–10 mIU/mL
DHEA-S	950.80 μg/dL	24–537 μg/dL
DHEA-S after dexamethasone suppression	221.20 μg/dL	24–537 μg/dL
		
		
		
		
		


FSH; Follicle-stimulating hormone, LH; Luteinizing hormone, PRL; Prolactin; PRGE; Progesterone, E2; Estradiol, T; Testosterone, hCG; Human chorionic gonadotropin and DHEA-S; Dehydroepiandrosterone sulfate.

**Table 3:** Levels of other adrenal secretions


Time	Level	Reference range

ALD	103.70 ng/dL	3.81–31.33 ng/dL
17-KS	46.4 mg/24 hours	10–25 mg/24 hours
17-OH	25.2 mg/24 hours	6–22 mg/24 hours
VMA	9.6 mg/24 hours	1.4–8 mg/24 hours


ALD; Aldosterone; 17-KS; 17-ketone steroids; 17-OH; 17-hydroxyl steroids and VMA; Vanilla almond acid.

We performed a genetic analysis of the patient, as well. DNA sequencing was performed according to the method of Zhang et al. (8).

Computed tomography (CT) and magnetic resonance imaging (MRI) were performed using an Aquilion16-slice spiral CT (Toshiba, Japan) and an ultra-high field 3.0T scans (Siemens, Germany), respectively, to examine the patient’s adrenal glands and testicles. Hematoxylin-eosin (H&E) staining and immunohistochemistry were performed to study the pathology of the TARTs.

**Fig.1:**
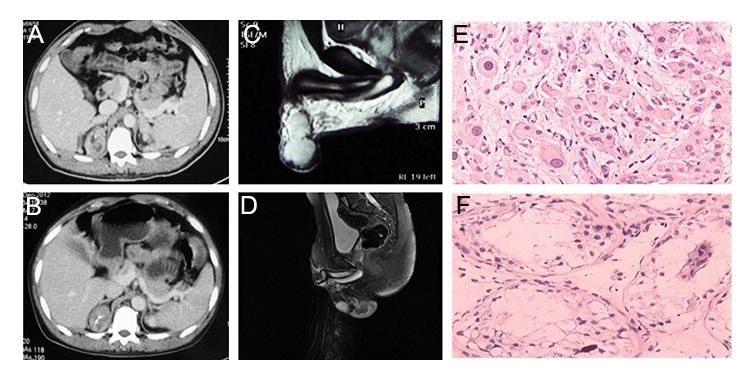
Multislice computed tomography, magnetic resonance imaging, and pathological examination of testicular adrenal rest tumors and testes. A. Mass with soft tissue density is noted on the right side of both adrenal glands; its maximum size is 4.5×3.7 cm, B. The adrenal masses are reduced in size following treatment), C. Both testes are enlarged, especially that on the left side, D. An obvious reduction in size of the masses on both testes is evident after treatment, E. A polygonal or circular eosinophilic cytoplasm is evident within the testicular tissue (H&E stain) and F. The spermatogenic cells in the seminiferous tubules are considerably diminished in size or absent (H&E stain).

The urine test results showed a decreased specific gravity (1.003), but liver and kidney function, routine blood test results, and the levels of tumor marker proteins were normal. DNA sequencing revealed a homozygous mutation (I2 g) on intron 2 of the patient’s *CYP21A2* gene. Multislice CT (non-contrastenhanced and contrast-enhanced) revealed a mass with a soft tissue density on the right side of both adrenal glands; its maximum size was 4.5×3.7 cm. The mass was irregularly shaped with a heterogeneous density, and exhibited a "fast-in and fast-out" pattern. MRI (non-contrast-enhanced and contrastenhanced) showed that both testes were irregularly enlarged, especially the left gonad. The mass within each testis had heterogeneous signal intensity and was surrounded by liquid signals. Several partitions were seen within the lesions. The testes themselves showed obvious and heterogeneous enhancement. Pathologically, the testes showed a polygonal or circular eosinophilic cytoplasm within the testicular tissue. Interstitial cells were in a cord-like arrangement, and lipid pigment was observed within the cytoplasm without Reinke crystals. Pathological investigation of the testicular tissue indicated that spermatogenic cells in the seminiferous tubules were considerably diminished or even absent. Sertoli cell hyperplasia was observed, but not typical spermatogenesis. Together, these imaging and clinical results suggested an initial diagnosis of adrenogenital syndrome, but the possibility of Leydig cell tumors was excluded (Fig .1).

On the basis of all the aforementioned results, the patient was diagnosed to have bilateral TARTs associated with CAH. Consequently, hydrocortisone was first administered for three months (20 mg, orally, twice daily) for treatment of the disease. However, his clinical symptoms did not improve. We then started the patient on dexamethasone replacement therapy (daily, oral dose of 0.75 mg). After six months of therapy, the patient’s skin lightened, and ultrasonography revealed gradual narrowing of his testicular nodules; the levels of sex hormones, ACTH, CO, and DHEA-S also normalized. After two years of treatment, his symptoms further alleviated, after which his oral dexamethasone dosage was reduced (0.5 mg, daily). Recent CT and MRI reexaminations showed shrinkage of the hyperplastic nodules on the adrenal glands and the testicular tumors. MRI showed that the soft tissue-density mass remained in the intramural, collateral branch of the right adrenal gland, but it had reduced in size. The mass in the left adrenal gland had disappeared, and bilateral adrenocortical hyperplasia had recurred. The effectiveness of the treatment was proven by the reduced size of the masses in both testes (Fig .1).

## Discussion

CAH comprises a group of autosomal recessive genetic disorders resulting from mutations in the genes involved in CO synthesis in the adrenal glands. Non-classic (delayed-type) CAH involves mild 21-OHD, resulting in mild clinical manifestations of hyperleydigism. In boys, these symptoms usually manifest as an abnormally large penis, advanced puberty or slightly accelerated growth, shorter stature, dark skin, spermatogenic dysfunction, or even infertility. The boundary between simple virilizing and non-classic CAH is difficult to define because the level of 17α-hydroxyprogesterone (17-OHP) varies across a continuum between mild and severe cases. Increased levels of 17-OHP are specific indicators of 21-OHDs. Based on clinical manifestations and the level of 17-OHP, a relatively accurate diagnosis of classic CAH can usually be made. When serum 17-OHP levels are approximately at normal levels and do not provide sufficient diagnostic information, an "ACTH stimulation test" is necessary. In our case, a whole body check of our patient revealed signs of hyperandrogenism, including skin pigmentation, testis/ adrenal hyperplasia, and precocious puberty. After determining ACTH and CO levels, we administered a “low-dose dexamethasone suppression test” and found the patient’s level of ACTH could be effectively restrained. Moreover, his levels of ACTH, urinary 17-KS, and DHEA-S, and serum PRGE (which can convert into 17-OHP) were all increased, but his CO level was reduced. These results excluded a hypothalamus pathogeny, because of which we considered that the patient’s symptoms were caused by an adrenal disease, such as CAH. Unfortunately, our hospital lacks the facility for detecting 17-OHP levels. Therefore, an ACTH stimulation test could not be conducted. In general, however, the patient’s clinical signs and metabolic features indicating simple virilizing CAH were relatively obvious, and a definite diagnosis was made after all of the available results, including DNA sequencing, we considered.

Pathologically, hyperplasia of the adrenal glands and accumulation of precursor material can lead to excessive synthesis of androgen; elevated levels of adrenal androgen may thereby inhibit gonadotropin levels. Therefore, determination of LH and FSH levels can indirectly provide an evaluation of a male CAH patient’s gonadal function. Compared to patients with low gonadotropin levels due to thyroid hypofunction, T levels of CAH patients are usually normal or only slightly reduced because of the elevated adrenal androgen levels (9). In our patient, the decreased levels of FSH and LH might have been associated with his prepubescence or his suggested pseudo-precocious puberty. Furthermore, the patient’s testosterone level was within the normal range, eliminating a low gonadotropin level caused by hypothyroidism.

In some cases, pseudo-precocious puberty may activate the hypothalamus-pituitary-gonadal axis, causing central precocious puberty. Large doses of androgens may also cause premature epiphyseal closure in patients. Our patient’s radiographic examination indicated that his bone age was more than three years older than his chronological age, suggesting the etiology of his short stature. In addition, pigmentation in his gums and external genitalia may have been due to weakened feedback inhibition involving ACTH and stimulating hormones that regulate melanin secretion.

The gene responsible for the production of 21- OH, in our patient, was composed of an inactive CYP21A1 (pseudogene) and an active *CYP21A2* (true gene). In humans, about 1/60 individuals carry a *CYP21A2* mutation, allowing genotyping to contribute to a precise diagnosis of CAH (10). DNA sequencing can clearly detect both heterozygous alleles in patients and may provide valuable guidance for genetic counseling and prenatal diagnoses. In our case, DNA sequencing revealed a homozygous mutation on intron 2 of the *CYP21A2* gene, leading to 21-OHD and adrenal insufficiencies. Such an intron 2 splice usually results in salt wasting CAH; however, the mutation may also be found in approximately 10% of cases of simple virilizing CAH, but almost never in patients with non-classic CAH.

Due to insufficient endocrine regulation, the compensatory secretion of pituitary ACTH may reach a sufficiently high level to cause hyperplasia of testicular adrenal-derived cells. The presence of abnormal, residual adrenal cells may lead to TARTs, but are not associated with malignant tumors. The immunohistochemical results also excluded a Leydig cell tumor diagnosis.

Claahsen-van der Grinten et al. (9) reported that ultrasonography provides the best detection and follow-up method for TARTs, especially for nonpalpable tumors. In imaging studies, the diameter of a TART is usually <2 cm, and it is surrounded by a hypoechoic area. The tumor may clog the seminiferous tubules, affecting testicular function, or even lead to infertility (1, 11). MRIs of the testicular tumors in the present case showed irregular nodules in both testes and a nonhomogeneous signal in the TARTs. Diaphragms were also present in the TARTs, surrounded by hypoechoic signals. Thus, we recommend ultrasonography as a regular follow-up method in such patients in order to avoid iatrogenic radiation and to minimize patient discomfort. However, when the patient’s condition changes, an MRI should be performed, as it contributes greatly to the disease diagnosis (12, 13).

Benvenga et al. (14) found LH receptors in TARTs and speculated that increased LH levels during puberty were an additional stimulus for the pathogenesis of TARTs. This might explain the increased incidence of TARTs in CAH patients during puberty or postpuberty. In clinical practice, testicular nodules are easily mistaken for Leydig cell tumors, because of which orchiectomy may be performed (15). Electron microscopic examinations have shown that TARTs are histologically similar to Leydig cell tumors, and that they involve the same steroid-secreting cells. However, TARTs do not contain the Reinke crystals observed in Leydig cell tumors (11). Up to 80% of CAH patients may exhibit TARTs in both testes, but only 3% of patients with interstitial cell tumors exhibit a TART in the same testis (9). Therefore, when CAH is accompanied by nodules in both testes, a TART diagnosis should be considered first.

Histological analysis indicated that the identification of the differences between atypical proliferation of steroid-hormone-secreting cells and rapid mitosis is an effective method for distinguishing benign TART from malignant testicular interstitial cell tumors. In addition, real time-polymerase chain reaction (real time-PCR) analysis of Leydig cell tumors results in unique gene products (14). In the present case, immunohistochemistry showed that the mass was vimentin (+), indicating that both testicular masses were derived from the mesenchyme. Additionally, the absence of Reinke crystals excluded the possibility of malignancy.

Adrenal hyperplasia accompanied by TART can highly increase the probability of low fertility in men. Although TART has no malignant features, the tumor near the testicular mediastinum may oppress seminiferous tubules and eventually decrease fertility (7). Reduced diameters of the seminiferous tubules, fibrosis, and hyalinosis may be observed in the testis, and the number of spermatogenic cells may decrease considerably. TART also has a paracrine role. Steroidal toxic substances produced by tumor cells may damage Leydig and germ cells (16), and thus indirectly disrupt reproductive hormone levels. In addition, excessive glucocorticoid replacement therapy may also affect the quality of semen and thus affect fertility. The inhibin B level in TART patients is most likely to affect spermatogenic function in infertile men (17, 18), but appropriate medical treatment may improve semen quality.

In the present case, spermatogenic cells of the patient reduced significantly owing to support cell proliferation. Glucocorticoid replacement therapy compensated for the lack of CO, while the secretion of ACTH was inhibited. Hydrocortisone is usually the drug of first choice in this treatment, although it has relatively weak efficacy; it has few side effects. We first administered hydrocortisone treatment to our patient for three months, but noted little improvement. Thus, we switched to dexamethasone, a relatively strong drug. After adequate drug treatment, the levels of the adrenal precursors and DHEA-S, which can cause hyperandrogenism, decreased, and gonadal hormone levels returned to normal. The growth and maturation of the testicular seminiferous tubules were restored, and testicular nodules either decreased in size or disappeared. Unfortunately, sperms were not detected in the semen of our patient during the three-year follow-up.

In some patients with hormone-insensitive CAH, accompanied by TARTs, the clinical symptoms may not improve after the administration of conservative hormone replacement therapy. In these cases, because of improvements in the general condition of the patients, as well as in their quality of life, fertility maintenance, and prevention of glucocorticoid and mineralocorticoid side effects (18), testicular tumor excision should be considered. Tiryaki et al. (19) reported 2 cases of hormone- sensitive adenomatous hyperplasia in which nodular enucleation was performed, and good long-term results were obtained; the nodules and metastasis did not recur. Walker’s testis-sparing surgery has also been found to be useful as the testes retained a satisfactory postoperative vascular flow, and tumor recurrence was not observed (20). However, enucleation of multiple nodules may be accompanied with an increased risk of testicular atrophy. Thus, for fertile TART patients, we recommend preoperative sperm-banking.

## Conclusion

We described a case of simple virilizing CAH with TARTs in a 15-year-old boy. Physicians should consider CAH, especially in patients with bilateral TARTs, especially since TARTs are rarely the presenting symptoms of CAH. The occurrence of TARTs may be related to the continuous production of ACTH in patients with delayed treatment for CAH. If a malignant tumor is ruled out, however, it is necessary to carefully determine the need for surgical removal of the tumor. Satisfactory curative effects may be obtained with appropriate glucocorticoid replacement therapy. Upon encountering such a case, early diagnosis and timely treatment are required to avoid adverse consequences caused by misdiagnoses and also to improve the patient’s quality of life, as much as possible.
